# Altered Crossover Distribution and Frequency in Spermatocytes of Infertile Men with Azoospermia

**DOI:** 10.1371/journal.pone.0156817

**Published:** 2016-06-06

**Authors:** He Ren, Kyle Ferguson, Gordon Kirkpatrick, Tanya Vinning, Victor Chow, Sai Ma

**Affiliations:** Department of Obstetrics and Gynaecology, University of British Columbia, Vancouver, B.C., Canada; University of California, San Francisco, UNITED STATES

## Abstract

During meiosis, homologous chromosomes pair to facilitate the exchange of DNA at crossover sites along the chromosomes. The frequency and distribution of crossover formation are tightly regulated to ensure the proper progression of meiosis. Using immunofluorescence techniques, our group and others have studied the meiotic proteins in spermatocytes of infertile men, showing that this population displays a reduced frequency of crossovers compared to fertile men. An insufficient number of crossovers is thought to promote chromosome missegregation, in which case the faulty cell may face meiotic arrest or contribute to the production of aneuploid sperm. Increasing evidence in model organisms has suggested that the distribution of crossovers may also be important for proper chromosome segregation. In normal males, crossovers are shown to be rare near centromeres and telomeres, while frequent in subtelomeric regions. Our study aims to characterize the crossover distribution in infertile men with non-obstructive (NOA) and obstructive azoospermia (OA) along chromosomes 13, 18 and 21. Eight of the 16 NOA men and five of the 21 OA men in our study displayed reduced crossover frequency compared to control fertile men. Seven NOA men and nine OA men showed altered crossover distributions on at least one of the chromosome arms studied compared to controls. We found that although both NOA and OA men displayed altered crossover distributions, NOA men may be at a higher risk of suffering both altered crossover frequencies and distributions compared to OA men. Our data also suggests that infertile men display an increase in crossover formation in regions where they are normally inhibited, specifically near centromeres and telomeres. Finally, we demonstrated a decrease in crossovers near subtelomeres, as well as increased average crossover distance to telomeres in infertile men. As telomere-guided mechanisms are speculated to play a role in crossover formation in subtelomeres, future studies linking crossover distribution with telomere integrity and sperm aneuploidy may provide new insight into the mechanisms underlying male infertility.

## Introduction

During the first meiotic division, a protein structure called the synaptonemal complex (SC) forms between the homologous chromosomes to facilitate the exchange of genetic material in a process known as recombination. Recombination is initiated with the formation of double strand breaks (DSBs) in the pre-leptotene stage of Prophase I, and completed during the pachytene stage where crossovers result [1]. The location of DSB formation, which DSBs are repaired as crossovers, and timing of DSB initiation can simultaneously affect the crossover landscape [2]. Other mechanisms thought to govern the distribution of DSBs include DNA and histone modifications as they alter the chromatin’s accessibility to proteins involved in the process [2].

Many studies have reported reduced rates of recombination in infertile men, which is thought to contribute to incorrect chromosome segregation during meiosis, and the subsequent production of aneuploid sperm with an extra chromosome (disomy) or missing chromosome (nullisomy) [3–7]. Our previous study found an inverse correlation between the frequency of sex chromosome recombination and XY disomy in the sperm [6]. These results suggest that increased errors in recombination may explain the elevated levels of aneuploid sperm observed in infertile men [6,8]. Aside from errors in the number of crossovers formed, studies on chiasma have suggested that infertile men may also display changes in crossover positions [9–12]. In a preliminary study, we used immunofluorescence techniques to directly visualize the distribution of crossovers on chromosomes 13, 18 and 21 in ten infertile men and five fertile men [5]. We reported two infertile men with normal crossover frequencies, but altered crossover distributions. Prior to our finding, only one infertile man was reported to possess a normal recombination rate, but altered crossover positions [11]. These limited studies suggest that infertile men may display altered crossover frequencies, crossover positions or both [5,11].

Although mutations in meiosis-specific genes are implicated in a small percentage of male infertility, the mechanism behind meiotic errors remain largely elusive [13]. A structure observed to be compromised in infertile men is the ribonucleoprotein cap of chromosome ends, known as the telomere [14]. Telomeres promote synapsis by forming a bouquet where they cluster and attach to the inner nuclear envelope [15–17]. Failed telomere attachment may result in perturbed DSB repair and meiotic cell arrest [18,19]. Although mice with compromised telomeres have been associated with impaired synapsis, recombination, and increased sperm aneuploidy rates [20–25], whether defective telomeres affect the crossover position is yet to be investigated. In normal males, an inhibition of crossovers near centromeres and the vicinity of telomeres have been observed [26,27], which is thought to drive proper chromosome segregation [28,29]. Studies have also indicated a high frequency of crossovers near subtelomeres [5,12,30]. In our preliminary work, two infertile men with altered crossover distributions showed increased crossover formation near centromeres, and decreased crossovers near subtelomeres [5]. In this study, we aimed to further investigate whether this population displays a trend of crossovers shifting toward the centromere. To achieve our aims, we analyzed the frequency and distribution of crossovers, as well as the distance from crossovers to telomeres on chromosomes 13, 18 and 21 in a large sample size (n = 65). Our findings may shed light on the consequences of altered crossover positions on spermatogenic arrest, and thus infertility, as well as increased incidences of sperm aneuploidy in infertile men.

## Materials and Methods

### Patient and tissue collection

Testicular tissue was collected from 37 infertile men seeking fertility treatment and 28 proven fertile men undergoing vasectomy reversals. Fertile men whose vasectomy lasted more than ten years were excluded from the study. Although fertile men whose reproductive tract have not been altered would make ideal controls, it is very unlikely to receive testicular tissue from this cohort. All men had normal 46,XY karyotypes, no microdeletions on the Y chromosome, and no cystic fibrosis transmembrane conductance regulator (CFTR) mutations. Sixteen of the infertile men were classified as non-obstructive azoospermia (NOA), and diagnosed with either hypospermatogenesis or maturation arrest. The other 21 men were classified as obstructive azoospermia (OA), where the pathological diagnosis showed normal spermatogenesis despite having no sperm in the semen. Synaptic defects, crossover frequencies and crossover distributions for five fertile men, and nine infertile men (Tables 1 and 2) were previously reported [5].

**Table 1:** Analysis of recombination and synaptic errors in control and NOA men.

	Number of cells	Mean rate (± SD) of genome-wide recombination	Proportion of cells with unsynapsed regions (%)
**Control men (*n* = 28)**			
Mean (95% CI)	1958	48.5 (47.6–49.4)	2.6 (1.5–3.8)
**Non-obstructive azoospermic men (*n* = 16)**			
NOA4	113	48.9 ± 3.5	0
NOA5	99	50.2 ± 5.3	0
NOA10*	51	**42.0 ± 4.7**^**a**^	**11.8**^**c**^
NOA13*	100	**34.0 ± 3.0**^**a**^	**14.0**^**c**^
NOA15*	61	**45.2 ± 3.5**^**a**^	1.6
NOA16*	53	**41.7 ± 4.0**^**a**^	1.9
NOA18*	100	49.9 ± 4.6	**14.0**^**c**^
NOA21	33	49.1 ± 3.8	**6.9**^**c**^
NOA22	100	48.8 ± 3.4	0
NOA23	100	**46.1 ± 3.1**^**b**^	**4.0**^**c**^
NOA24	53	**46.4** **±** **3.4**^**b**^	0
NOA25	35	**41.4** **±** **3.7**^**a**^	3.3
NOA26	100	53.8 ± 5.7	2.0
NOA27	86	52.1 ± 3.9	1.1
NOA28	101	46.6 ± 6.6	3.0
NOA29	45	**40.9** **±** **3.5**^**a**^	**7.1**^**c**^
Mean (95% CI)		46.2 (43.8–48.6)	5.0 (2.46–7.5)

^a^P < 0.001

^b^P < 0.05

recombination significantly reduced when compared with controls, Mann-Whitney Test.

^c^Proportions of cell with unsynapsed regions were considered significantly different from controls if they were beyond the 95% CI of the control group

*Previously reported by Ferguson et al. (2009)

**Table 2:** Analysis of recombination and synaptic errors in control and OA men.

	Number of cells	Mean rate (± SD) of genome-wide recombination	Proportion of cells with unsynapsed regions (%)
**Control men (*n* = 28)**			
Mean (95% CI)	1958	48.5 (47.6–49.5)	2.6 (1.5–3.8)
**Obstructive azoospermic men (*n* = 21)**			
OA6	52	48.2 ± 3.7	0
OA7*	100	47.6 ± 4.5	3.0
OA9*	100	47.4 ± 4.5	**26.0**^**c**^
OA11*	73	**46.5 ± 3.8**^**b**^	**8.2**^**c**^
OA14*	100	**42.1 ± 4.7**^**a**^	3.0
OA19	100	53.2 ± 4.2	**19.0**^**c**^
OA20	100	48.8 ± 3.9	3.0
OA21	113	47.4 ± 4.4	0.9
OA22	63	51.0 ± 5.6	**15.9**^**c**^
OA24	75	50.1 ± 4.8	**20.8**^**c**^
OA25	53	54.9 + 4.4	1.0
OA26	28	46.0 ± 4.7	0
OA27	32	46.8 ± 7.1	3.1
OA28	58	50.8 ± 3.5	**10.3**^**c**^
OA29	40	**42.5** **±** **3.1**^**a**^	3.0
OA30	41	47.0 ± 4.5	3.3
OA31	39	**43.0** **±** **3.2**^**a**^	**8.2**^**c**^
OA32	42	48.5 ± 4.9	2.4
OA33	45	50.5 ± 6.1	2.1
OA34	41	**46.2** **±** **4.2**^**b**^	**5.2**^**c**^
OA35	99	50.7 ± 6.2	3.0
Mean (95% CI)		48.0 (46.6–49.4)	6.7 (3.5–9.9)

^a^P < 0.001

^b^P < 0.05, recombination significantly reduced when compared with controls, Mann-Whitney Test.

^c^Proportions of cell with unsynapsed regions were considered significantly different from controls if they were beyond the 95% CI of the control group

*Previously reported by Ferguson et al. (2009)

### Meiotic analyses

**Fig 1:**
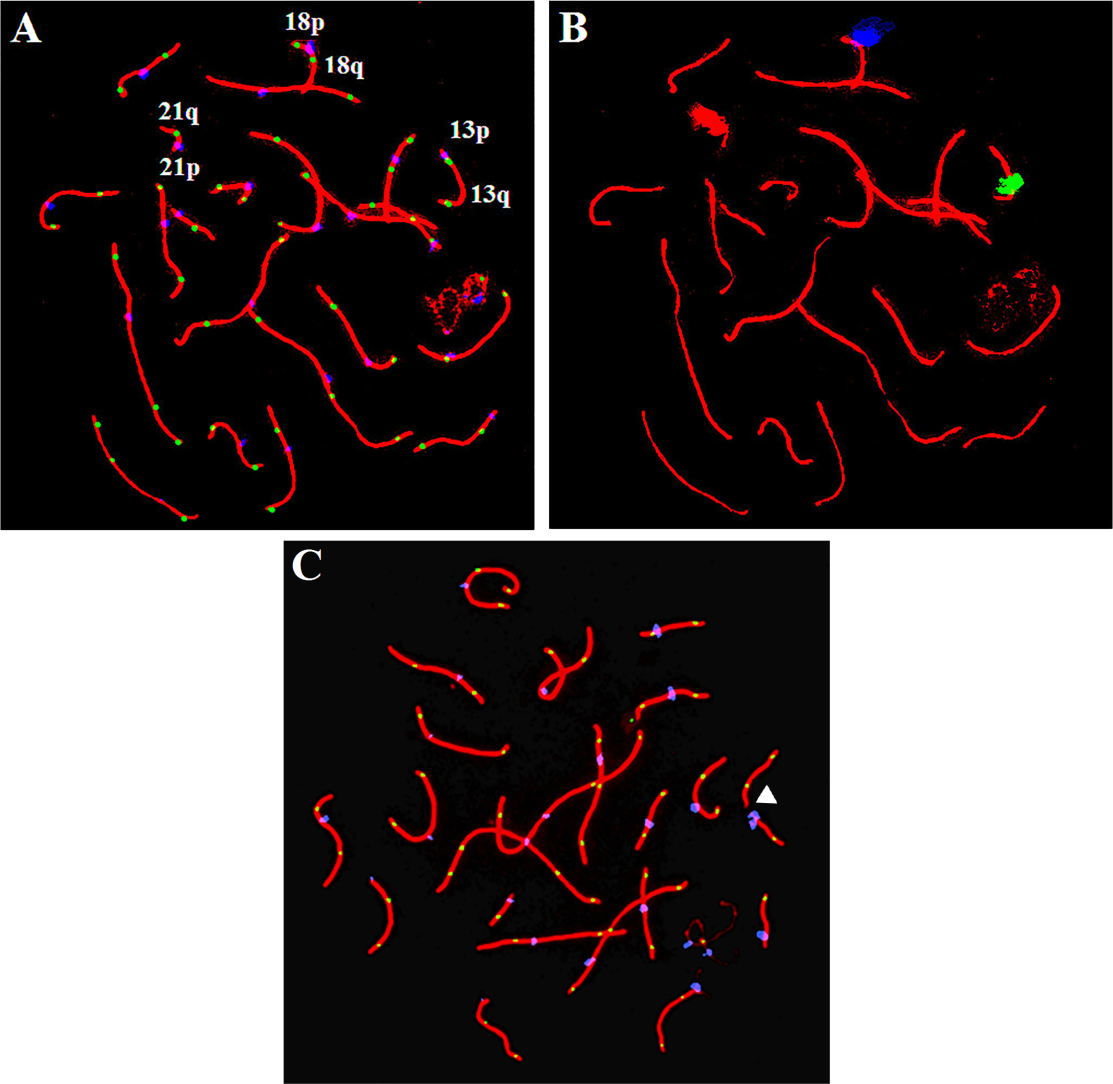
Immunofluorescence and FISH analysis of pachytene spermatocytes. (A) Spermatocytes were immunostained using antibodies against SYCP3/SYCP1, MLH1 and CREST to visualize the SC (red), crossover sites (green) and centromeres (blue). A spermatocyte with 45 crossovers from patient OA20 is shown. Although patient OA20 displayed normal rates of recombination, the crossover distribution on chromosome 18 was altered. We observed an increase in crossovers near the centromere and telomere on 18q and 18p, respectively. (B) Subsequent FISH was performed to identify chromosomes 13 (green, LSI 13), 18 (blue, CEP 18) and 21 (red, LSI 21) in the previously immunostained spermatocytes. (C) A spermatocyte from patient OA19 is shown. Although the patient showed normal rates of recombination, we observed an increased rate of synaptic errors compared to controls (Table 1). The unsynapsed region along a bivalent is indicated by the white arrow, where there is an absence of staining for SYCP3/SYCP1.

Testicular tissue was processed according to a previously described protocol [4]. Spermatocytes were immunostained with antibodies against SYCP3/SYCP1, MLH1 and CREST antisera in order to visualize the SC, crossover sites and centromeres respectively. Immunostained spermatocytes were examined using a Zeiss Axioplan epifluorescent microscope equipped with the appropriate filters. Cytovision V2.81 Image Analysis software (Applied Imaging International, San Jose, CA, USA) was used to capture the SYCP3/SYCP1, MLH1 and CREST signals on the pachytene spermatocytes (Fig 1A). The number of MLH1 foci per cell, frequency of synaptic errors, and cell coordinates were recorded.

**Fig 2:**
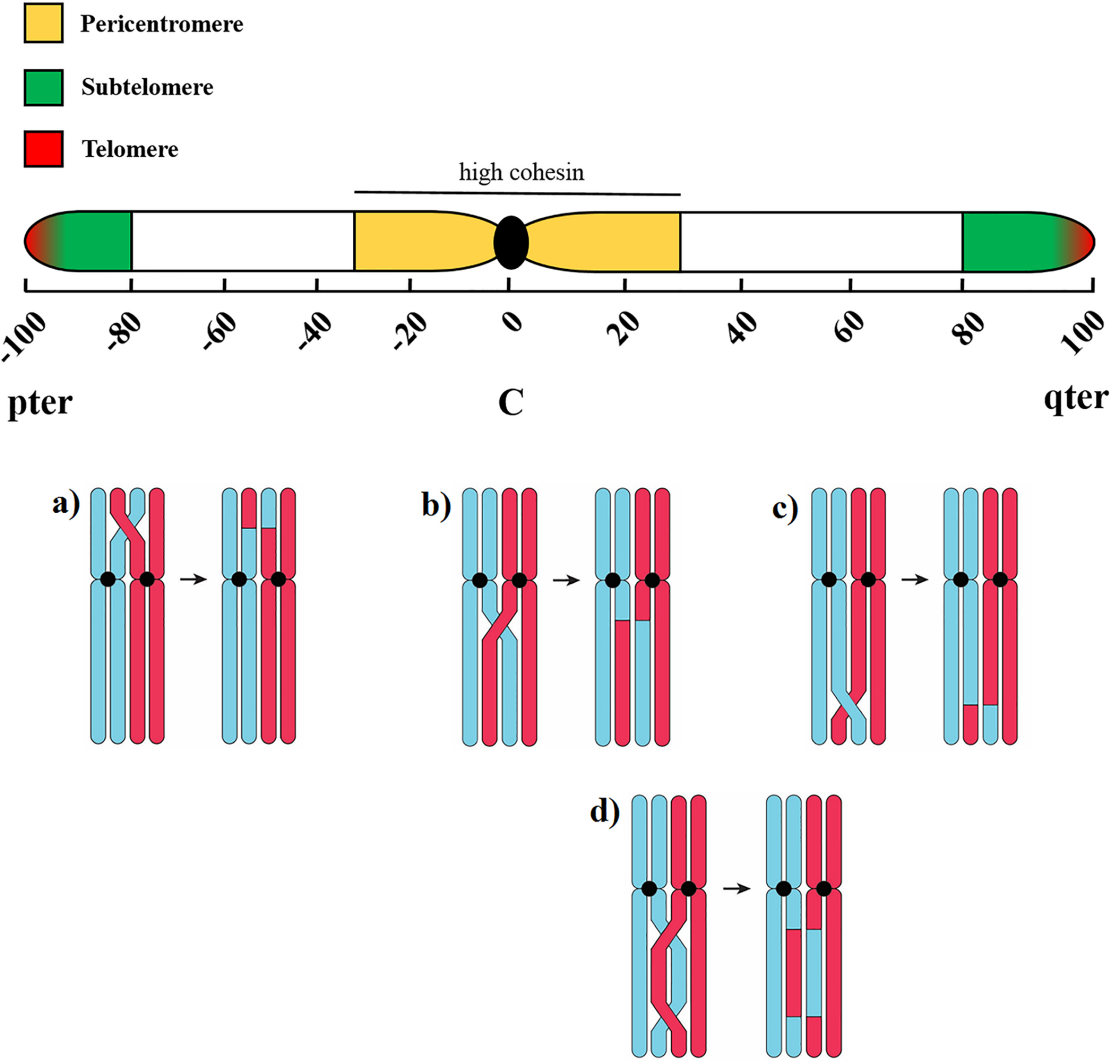
Diagram depicting meiotic crossovers in regions along a chromosome. The p and q arms of the chromosome are divided into 10% intervals, with the centromere (C) at 0%, and telomeres at 100%. The subtelomere is shaded in the 80–100% intervals. The pericentromere, the region surrounding the centromere is shaded in the 10–30% intervals. This region attracts high levels of cohesin which are protein complexes that hold sister chromatids together until they separate during meiosis. Crossover formation in each region of the chromosome, and the resulting recombinant chromosomes are illustrated: a) a single crossover near the telomere on the p-arm; b) a single crossover near the centromere on the q-arm; c) a single crossover near the telomere on the q-arm; d) double crossovers on the q-arm.

As the most common types of autosomal trisomy in live births are trisomy 21, 18 and 13, we chose to study the meiotic patterns and sperm aneuploidy of these specific chromosomes in the infertile men and controls. We are curious as to whether abnormal meiotic behaviors may lead to an increased risk of sperm aneuploidy for these chromosomes in certain sub-groups of infertile men. To study this pattern, FISH was performed on the previously immunostained slide according to the methods reported by Ma *et al*. [4]. A probe mixture of LSI 13 (green), CEP 18 (aqua) and LSI 21 (red) (Vysis Inc., Downers Grove, IL, USA) was used to identify chromosomes 13, 18 and 21 (Fig 1B). The crossover distribution, represented by the distribution of MLH1 foci, and SC lengths were measured using the image analysis software Micromeasure V3.3, available at: sites.biology.colostate.edu/MicroMeasure/ [31]. The SC arms of the chromosomes 13, 18 and 21 were divided into 10% intervals, with the centromeres at 0% and telomeres at 100% (Fig 2). The frequency of MLH1 foci in each interval was calculated (Fig 2). As the number of crossovers along a chromosome significantly influences their distribution [5], we separately analyzed the crossover distributions on chromosome 13 and 18 bivalents with single and double crossovers (Fig 2). The absolute distance between crossovers and telomeres was measured for each arm of chromosomes 13, 18 and 21, and then divided by the length of the SC arm in order to express the distance as a percentage.

### Statistical analyses

The Mann-Whitney test was used to compare the mean rate of genome-wide recombination between individual infertile men and the control group. The frequency of synaptic errors in the infertile men were considered significantly different if they were beyond the 95% confidence interval of the control group. The χ^2^ test with two degrees of freedom was used to compare the frequencies of crossovers on chromosomes 13 and 18, while a χ^2^ test with one degree of freedom was used to compare the crossover frequencies on chromosome 21 between individual infertile men and the control group. We used a χ^2^ test with 243 degrees of freedom to compare the crossover distribution within the control group and found no significant differences for any of the chromosome arms studied. Thus, the control men were pooled and a χ^2^ test with nine degrees of freedom was used to compare the crossover distribution between individual infertile men and the control group, as well as between the NOA/OA group and control group. The Fisher test was used to compare the crossover frequencies in each 10% interval between individual infertile men and the control group, as well as between the NOA/OA group and control group. The Mann-Whitney test was used to compare the average crossover distance to telomeres between the NOA/OA group and the control group. P < 0.05 was considered statistically significant. Written informed consent from patients, and ethical approval from the University of British Columbia Ethics Committee were obtained before initiating this study.

## Results

### Frequency of crossovers and synaptic errors

Eight of the 16 NOA men and five of the 21 OA men in this study displayed a reduced genome-wide recombination rate compared to controls (Tables 1 and 2). One of the NOA men showed altered crossover frequencies on chromosome 18, two men showed alterations on chromosome 21, and five men showed alterations on more than one chromosomes studied (Table 3). In the OA population, three men displayed altered crossover frequencies on chromosome 18, four men displayed alterations on chromosome 21, and two men showed alterations on more than one chromosomes studied (Table 4). With regards to homologous chromosome synapsis, although some spermatocytes in the control men showed synaptic errors, these defects were significantly more frequent in the spermatocytes of infertile men. In fact, six NOA and eight OA men showed an increased frequency of synaptic errors compared to controls (Tables 1 and 2). A representative spermatocyte from patient OA19 with a synaptic defect in the form of a gap on a bivalent is shown in Fig 1C.

**Table 3:** Analysis of crossover frequencies on chromosome 13, 18 and 21 in control and NOA men.

		Chromosome 13			Chromosome 18			Chromosome 21		
	Number of Cells	0 foci	1 focus	≥ 2 foci	0 foci	1 focus	≥ 2 foci	0 foci	1 focus	≥ 2 foci
**Control men (*n* = 28)**										
Total	1740	0.4% (7)	11.4% (198)	88.2% (1535)	0.1% (2)	25.0% (435)	74.9% (1303)	0.5% (9)	99.2% (1726)	0.3% (5)
**Non-obstructive azoospermic men (*n* = 16)**										
NOA4	100	0% (0)	19.0% (19)	81.0% (81)	0% (0)	32.0% (32)	68.0% (68)	0% (0)	100% (100)	0% (0)
NOA5	90	0% (0)	15.6% (14)	84.4% (76)	0% (0)	14.4% (13)	85.6% (77)	0% (0)	100% (90)	0% (0)
NOA10	45	**0% (0)**^**b**^	**31.0% (14)**^**b**^	**69.0% (31)**^**b**^	**2% (1)**^**b**^	**38% (17)**^**b**^	**60% (27)**^**b**^	**4.4% (2)**^**b**^	**95.6% (43)**^**b**^	**0% (0)**^**b**^
NOA13	92	**3.3% (3)**^**a**^	**84.4% (79)**^**a**^	**12.0% (11)**^**a**^	**1.1% (1)**^**a**^	**91.2% (84)**^**a**^	**7.6% (7)**^**a**^	**4.3% (4)**^**b**^	**95.7% (88)**^**b**^	**0% (0)**^**b**^
NOA15	39	0% (0)	20.5% (8)	79.5% (31)	0% (0)	38.5% (15)	61.5% (24)	**5.1% (2)**^**b**^	**94.9% (37)**^**b**^	**0% (0)**^**b**^
NOA16	26	**0% (0)**^**a**^	**65.4% (17)**^**a**^	**30.8% (8)**^**a**^	**0% (0)**^**c**^	**46.2% (12)**^**c**^	**53.8% (14)**^**c**^	0% (0)	100% (26)	0% (0)
NOA18	88	0% (0)	6.8% (6)	93.2% (82)	0% (0)	29.5% (26)	70.5% (62)	0% (0)	100% (88)	0% (0)
NOA21	30	0% (0)	16.7% (5)	83.3% (25)	0% (0)	33.3% (10)	66.7% (20)	3.3% (1)	96.7% (29)	0% (0)
NOA22	90	0% (0)	8.9% (8)	91.1% (82)	1.1% (1)	26.7% (24)	72.2% (65)	1.1% (1)	98.9% (89)	0% (0)
NOA23	92	0% (0)	10.9% (10)	89.1% (82)	**2.2% (2)**^**a**^	**85.9% (79)**^**a**^	**12.0% (11)**^**a**^	2.2% (2)	97.8% (90)	0% (0)
NOA24	50	0% (0)	20.0% (10)	80.0% (40)	0% (0)	18.0% (9)	82.0% (41)	**14.0% (7)**^**b**^	**86.0% (43)**^**b**^	**0% (0)**^**b**^
NOA25	30	**0% (0)**^**a**^	**83.3% (25)**^**a**^	**16.7% (5)**^**a**^	**0% (0)**^**b**^	**60.0% (18)**^**a**^	**40.0% (12)**^**b**^	**6.7% (2)**^**b**^	**93.3% (28)**^**b**^	**0% (0)**^**b**^
NOA26	88	0% (0)	8.0% (7)	92.0% (81)	0% (0)	10.2% (9)	89.8% (79)	0% (0)	100% (88)	0% (0)
NOA27	57	0% (0)	5.3% (3)	94.7% (54)	0% (0)	19.3% (11)	80.7% (46)	0% (0)	100% (57)	0% (0)
NOA28	95	0% (0)	10.5% (10)	89.5% (85)	0% (0)	16.8% (16)	83.1% (79)	0% (0)	100% (95)	0% (0)
NOA29	40	**2.5% (1)**^**a**^	**72.5% (33)**^**a**^	**15.0% (6)**^**a**^	**0% (0)**^**b**^	**57.5% (23)**^**b**^	**42.5% (17)**^**b**^	**5.0% (2)**^**b**^	**95.0% (38)**^**b**^	**0% (0)**^**b**^
Total	1052	**0.4% (4)**^**b**^	**25.5% (268)**^**b**^	**74.1% (780)**^**b**^	**0.5% (5)**^**b**^	**37.8% (398)**^**b**^	**61.7% (649)**^**b**^	2.2% (23)	97.8% (1029)	0% (0)

^a^P < 0.0001, χ^2^ test with two degrees of freedom

^b^P < 0.01, χ^2^ test with two degrees of freedom

^c^P < 0.05, χ^2^ test with two degrees of freedom

**Table 4:** Analysis of crossover frequencies on chromosome 13, 18 and 21 in control and OA men.

		Chromosome 13			Chromosome 18			Chromosome 21		
	Number of Cells	0 foci	1 focus	≥ 2 foci	0 foci	1 focus	≥ 2 foci	0 foci	1 focus	≥ 2 foci
**Control men (*n* = 28)**										
Total	1740	0.4% (7)	11.4% (198)	88.2% (1535)	0.1% (2)	25.0% (435)	74.9% (1303)	0.5% (9)	99.2% (1726)	0.3% (5)
**Obstructive azoospermic men (*n* = 21)**										
OA6	52	0% (0)	17.3% (9)	82.7% (43)	0% (0)	25.0% (13)	75.0% (39)	1.9% (1)	98.1% (51)	0% (0)
OA7	97	0% (0)	17.5% (17)	82.5% (80)	0% (0)	33.0% (32)	67.0% (65)	0% (0)	100% (97)	0% (0)
OA9	66	0% (0)	12.1% (10)	84.8% (56)	1.6% (1)	18.2% (12)	77.3% (53)	0% (0)	100% (66)	0% (0)
OA11	66	0% (0)	19.7% (13)	80.3% (53)	1.5% (1)	24.2% (16)	74.2% (49)	1.5% (1)	98.5% (65)	0% (0)
OA14	47	0% (0)	23.4% (11)	76.5% (36)	**0% (0)**^**b**^	**44.7% (21)**^**b**^	**55.3% (26)**^**b**^	2.1% (1)	97.9% (46)	0% (0)
OA19	62	0% (0)	8.06% (5)	91.9% (57)	0% (0)	17.7% (11)	82.3% (51)	**0% (0)**^**a**^	**91.9% (57)**^**a**^	**8.06% (5)**^**a**^
OA20	56	0% (0)	5.4% (3)	94.6% (53)	0% (0)	26.8% (15)	73.2% (41)	**8.9% (5)**^**a**^	**89.3% (50)**^**a**^	**1.8% (1)**^**a**^
OA21	99	0% (0)	20.2% (20)	79.8% (79)	**0% (0)**^**b**^	**44.9% (44)**^**b**^	**55.1% (55)**^**b**^	2.0% (2)	98.0% (97)	0% (0)
OA22	45	0% (0)	15.6% (7)	84.4% (38)	0% (0)	22.2% (10)	77.8% (35)	0% (0)	100% (45)	0% (0)
OA24	55	0% (0)	16.4% (9)	83.6% (46)	0% (0)	16.4% (9)	83.6% (46)	0% (0)	100% (55)	0% (0)
OA25	50	0% (0)	18.0% (9)	82.0% (41)	0% (0)	22.0% (11)	78.0% (39)	0% (0)	100% (50)	0% (0)
OA26	20	0% (0)	10.0% (2)	90.0% (18)	**0% (0)**^**c**^	**45.0% (9)**^**c**^	**55.0% (11)**^**c**^	**15.0% (3)**^**a**^	**85.0% (17)**^**a**^	**0% (0)**^**a**^
OA27	29	3.4% (1)	13.7% (4)	82.7% (24)	0% (0)	24.1% (7)	75.9% (22)	**10.3% (3)**^**a**^	**86.2% (25)**^**a**^	**3.4% (1)**^**a**^
OA28	49	0% (0)	8.2% (4)	91.8% (45)	0% (0)	18.4% (9)	81.6% (40)	0% (0)	100% (49)	0% (0)
OA29	35	0% (0)	8.6% (3)	91.4% (32)	0% (0)	28.5% (10)	71.4% (25)	0% (0)	100% (35)	0% (0)
OA30	40	0% (0)	5.0% (2)	95.0% (38)	0% (0)	15.0% (6)	85.0% (34)	2.5% (1)	97.5% (39)	0% (0)
OA31	35	0% (0)	14.3% (5)	85.7% (30)	**0% (0)**^**b**^	**51.4% (18)**^**b**^	**48.5% (17)**^**b**^	2.9% (1)	97.1% (34)	0% (0)
OA32	40	0% (0)	25.0% (10)	75.5% (30)	0% (0)	20.0% (8)	80.0% (32)	2.5% (1)	97.5% (39)	0% (0)
OA33	43	0% (0)	14.0% (6)	86.0% (37)	0% (0)	25.6% (11)	74.4% (32)	**14.0% (6)**^**a**^	**86.0% (37)**^**a**^	**0% (0)**^**a**^
OA34	41	**0% (0)**^**b**^	**31.7% (13)**^**b**^	**68.3% (28)**^**b**^	**0% (0)**^**b**^	**19.5% (8)**^**b**^	**80.5% (33)**^**b**^	**0% (0)**^**b**^	**95.1% (39)**^**b**^	**4.9% (2)**^**b**^
OA35	67	0% (0)	10.45% (7)	89.6% (60)	0% (0)	11.9% (8)	88.1% (59)	1.5% (1)	98.5% (66)	0% (0)
**Total**	1094	0.01% (1)	13.6% (149)	86.3% (944)	0.2% (2)	26.3% (288)	73.5% (804)	1.9% (21)	97.3% (1064)	0.8% (9)

^a^P < 0.0001, χ^2^ test with two degrees of freedom

^b^P < 0.01, χ^2^ test with two degrees of freedom

^c^P < 0.05, χ^2^ test with two degrees of freedom

### Distribution of crossovers in fertile men

**Fig 3:**
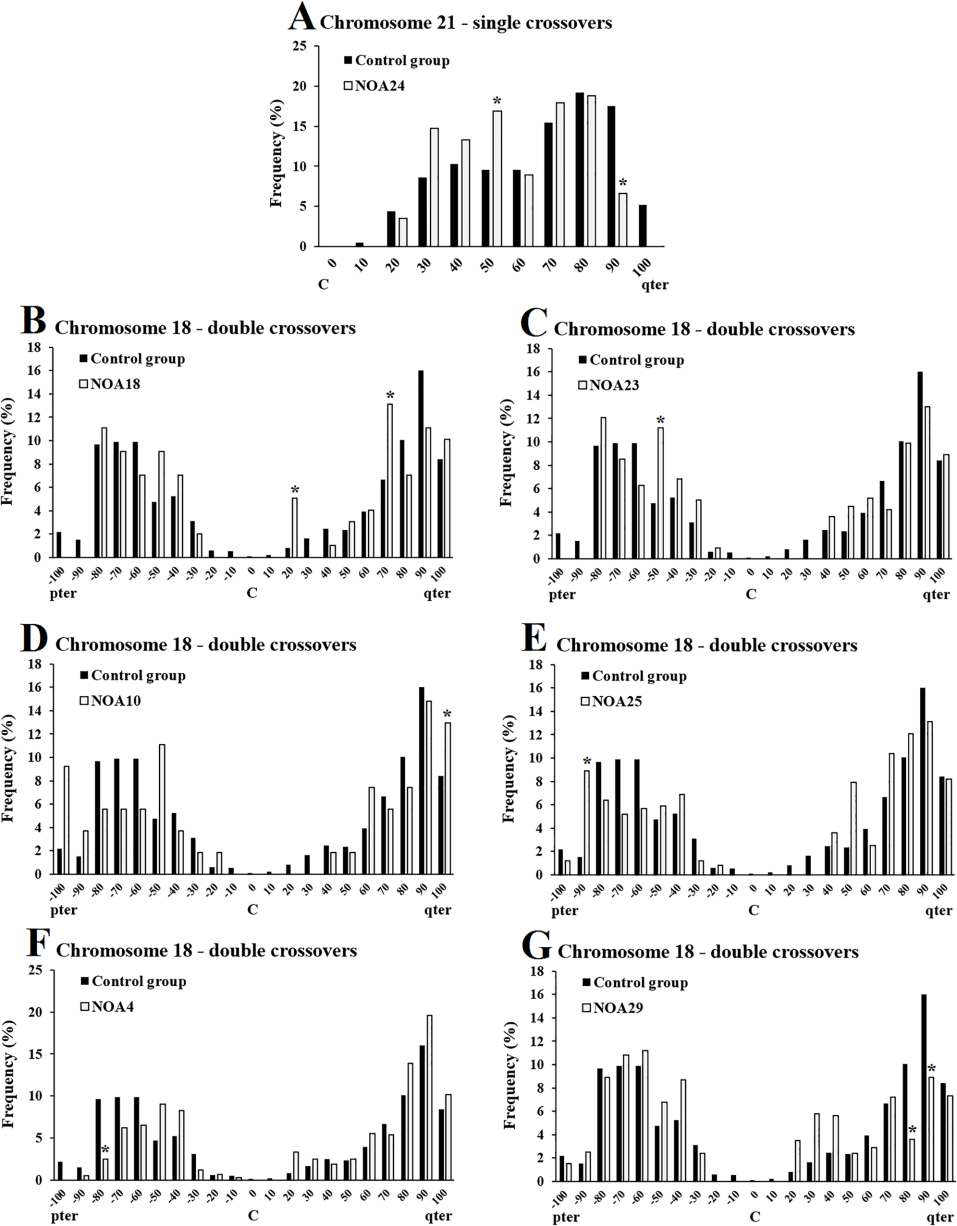
Chromosomes 21 and 18 displaying altered crossover distributions in NOA men. Chromosome arms were divided into 10% intervals, and the crossover frequency in each interval was calculated. The Y-axis represents the frequency of crossovers in each interval. The X-axis represents the relative crossover position from the centromere with the values representing the upper limit of each interval. The centromere is labeled ‘C’ with the p-arm to the left and q-arm to the right. As crossovers in the p-arm of chromosome 21 are extremely rare, the p-arm is not shown. The black bars indicate the control group and the white bars indicate the individual NOA man. The crossover frequencies in each interval were compared to the control group and significant differences are indicated by asterisks (P < 0.05, Fisher test).

**Fig 4:**
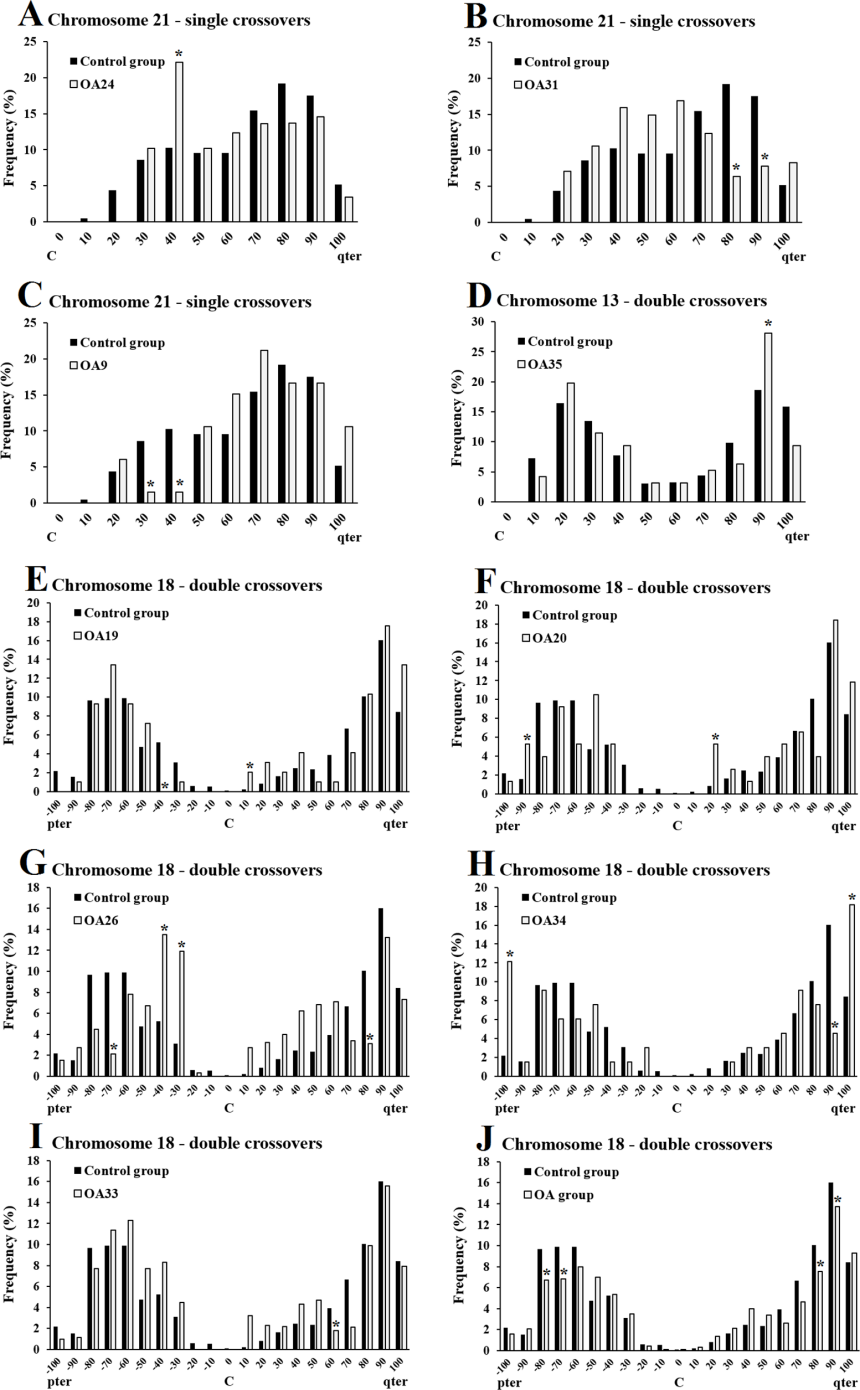
Chromosomes 21, 13 and 18 displaying altered crossover distributions in OA men. The Y-axis represents the frequency of crossovers in each interval. The X-axis represents the relative crossover position from the centromere with the values representing the upper limit of each interval. As crossovers in the p-arm of chromosomes 13 and 21 are extremely rare, the p-arms are not shown. The black bars indicate the control group and the white bars indicate the individual OA man (A-I) and pooled OA group (J). The crossover frequencies in each interval were compared to the control group and significant differences are indicated by asterisks (P < 0.05, Fisher test).

In the control population, single crossovers on chromosome 21q were most frequently located near subtelomeres, at relative distances to the centromere of 70–90% (denoted as intervals) as shown in Fig 3A. Similarly, double crossovers were most commonly found near subtelomeres on chromosome 18, where crossover frequency was highest at the 60–80% intervals on 18p, and 80–90% intervals on 18q (Fig 3B). Furthermore, crossover frequencies were low (< 5.0%) near the centromere on both arms of chromosome 18, as well as near the vicinity of the telomere on 18p (Fig 3B). In contrast, the pattern of crossover distribution was different on 13q, where crossovers were frequently located near the centromere and telomere (Fig 4D). In fact, crossover frequency was lowest (<5.0%) around the middle of 13q, at the 50–70% intervals (Fig 4D).

### Distribution of crossovers in NOA men

Compared to the control group, seven of the 16 NOA men displayed altered crossover distributions on at least one of the chromosome arms studied (P < 0.05, χ^2^ test, Fig 3). NOA24 displayed an altered crossover distribution on 21q with single crossovers, where there was an increase in crossovers near the middle of 21q (50% interval), and decrease in crossovers at the subtelomere (Fig 3A). In terms of chromosome 13, none of the NOA men showed an altered crossover distribution compared to controls (P > 0.05, χ^2^ test).

Six NOA men displayed altered crossover distributions on chromosome 18 with double crossovers compared to controls (Fig 3B–3G). NOA18 showed an increase in crossovers near the centromere and subtelomere (Fig 3B), while two men exhibited an increase in crossovers near the vicinity of the telomeres, at the 90–100% intervals (Fig 3D and 3E). In contrast, two NOA men showed a decrease in crossovers near subtelomeres (Fig 3F and 3G). NOA23 displayed an increase in crossovers at the 50% interval (Fig 3C). The pooled NOA group (n = 16) did not show significantly altered crossover distributions on any of the chromosomes studied (P > 0.05, χ^2^ test).

### Distribution of crossovers in OA men

Nine of the 21 OA men displayed altered crossover distributions on at least one of the chromosome arms studied compared to the control group (P < 0.05, χ^2^ test, Fig 4). Three of these men displayed altered crossover distributions on 21q with single crossovers (Fig 4). OA24 displayed an increase in crossovers near the centromere (Fig 4A), while OA31 displayed a decrease in crossovers near the subtelomere (Fig 4B). OA9 showed a decrease in crossovers near the centromere (Fig 4C). As for 13q with double crossovers, only OA35 displayed an altered crossover distribution (P < 0.05, χ^2^ test), where there was an increase in crossovers near the telomere at the 90% interval (Fig 4D).

Compared to the control group, five OA men displayed altered crossover distributions on either arms of chromosome 18 with double crossovers (Fig 4E–4J). Three men displayed an increase in crossovers near the centromere (Fig 4E–4G). Two men displayed an increase in crossovers near telomeres (Fig 4F and 4H). Two men showed a decrease in crossovers near subtelomeres (Fig 4G and 4H). OA33 showed a decrease in crossovers near the middle of 18q at the 60% interval (Fig 4I). The pooled OA group (n = 21) displayed an altered crossover distribution (P < 0.05, χ^2^ test) with a decrease in crossovers near subtelomeres on 18p and 18q (Fig 4J).

### Crossover distance to telomere

**Fig 5:**
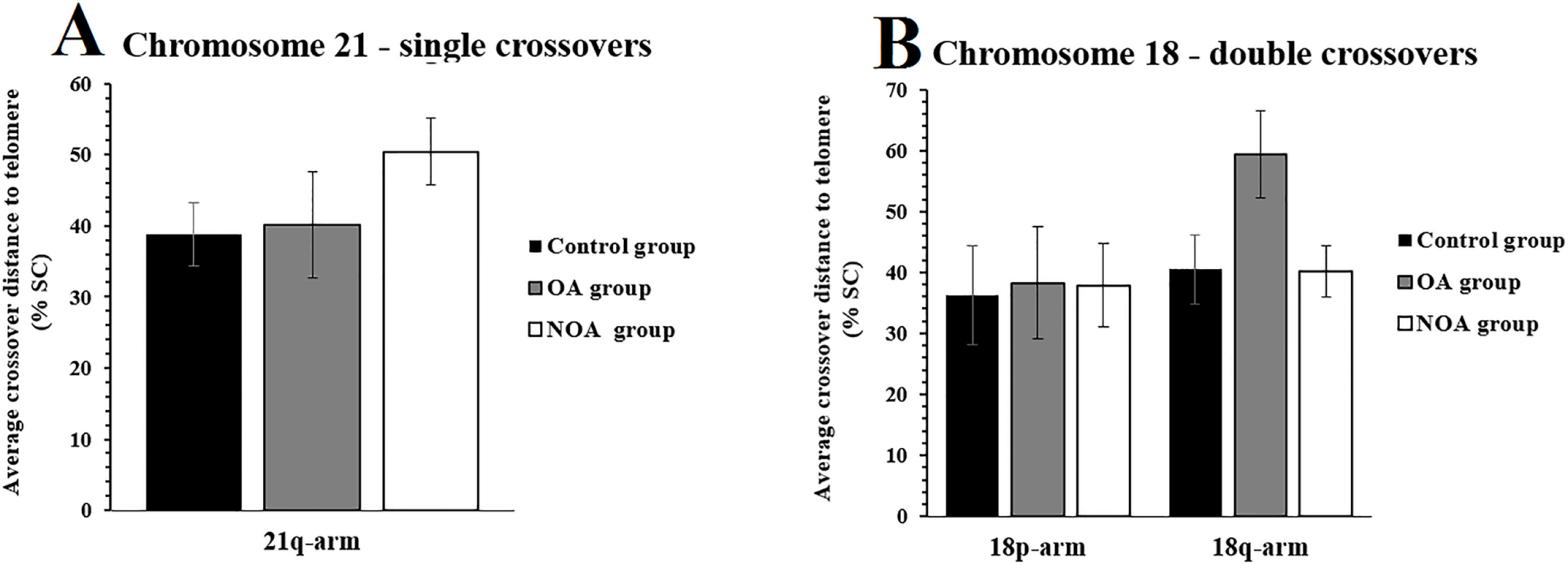
Average crossover distance to telomere (±SD) on chromosomes 18 and 21 in infertile and control men. The absolute distance between crossover and telomere was divided by the total SC arm length in order to express the distance as a percentage. The Y-axis represents the mean distance between crossovers to the telomeres on the chromosome arms 18q-arm, 18p-arm and 21q-arm. The black bars indicate the control group, the gray bars indicate the OA group and the white bars indicate the NOA group. The average distances between crossovers to telomeres were compared to the control group and significant differences are indicated by asterisks (P < 0.05, Mann-Whitney test).

On 21q, the NOA group displayed an increased average distance between crossover and telomere compared to controls, measured as a percentage of the total SC length (50.4%±4.7 vs. 38.8%±4.5, P < 0.05, Mann-Whitney test, Fig 5A). The OA group did not show any significance in this aspect compared to the control group (Fig 5A). Regarding chromosome 18, neither the NOA nor OA group showed a significant difference in mean crossover distance to telomere on 18p when compared to controls (Fig 5B). Nevertheless, the OA group showed an increased average crossover distance to telomere compared to controls on 18q (59.4%±7.2 vs. 40.5%±5.7, P < 0.05, Mann-Whitney test, Fig 5B). However, the NOA group did not show such significance compared to controls (Fig 5B). Neither the NOA nor OA group showed significantly different average distances between crossover and telomere on 13q compared to controls (P > 0.05, Mann-Whitney test).

### Crossover frequency in relation to crossover distribution

We observed two infertile men (NOA4 and OA35) with normal global and chromosome-specific recombination, who nevertheless displayed altered crossover distributions (Figs 3F and 4D). Six of the nine OA men with altered crossover distributions had either reduced global or chromosome-specific recombination rates. Only two OA men displayed altered crossover frequencies and distributions on the same chromosome. On the other hand, five of the eight NOA men with altered crossover distributions displayed reduced crossover frequencies on the same chromosome, as well as reduced global recombination rates.

## Discussion

In a preliminary study, we analyzed the crossover frequency and distribution on chromosomes 13, 18 and 21 in six NOA and four OA men [5]. With a larger sample size, our present study further investigates the crossover distributions of individual infertile men, as well as pooled NOA and OA groups. Moreover, we report the first study to examine the average distance from crossovers to telomeres on chromosomes 13, 18 and 21, with aims of identifying a shift in crossovers in infertile men. Our data concludes that infertile men may display alterations in the distribution of crossovers, where an increase in crossovers near the centromere and telomere, and decrease in crossovers near the subtelomere was observed. Furthermore, there may be inherently different risks for NOA and OA men for possessing alterations in either the frequency of crossovers, distribution of crossovers, or both, likely due to distinct underlying mechanisms.

### Increased crossovers near centromeres and telomeres in infertile men

In normal males, the distal regions of the chromosomes are largely composed of euchromatin and much more susceptible to recombination than the heterochromatin rich regions near the centromeres [2]. Analysis of DSBs in *Saccharomyces cerevisiae* revealed significant DSB formation near the centromeres, but an inhibition of crossover formation during DSB repair [29]. The inhibitory mechanism near the centromeres help ensure faithfulness during meiosis, where crossovers at these regions may lead to precocious sister chromatid segregation at meiosis II due to interference with centromeric cohesins [28,29]. In our study, the distribution of crossovers was altered in 7 NOA and 9 OA men when compared to the control group. Out of these 16 infertile men, five men displayed an increase in crossover formation near centromeres on one of the chromosomes studied. Although this shift of crossovers toward the centromere was previously observed in three infertile men [5,11], this is the first report in a group of NOA and OA men. Interestingly, we observed a shift of crossovers toward the centromere on 21q in two infertile men (NOA24 and OA24). Previously, human oocytes with increased frequencies of crossovers near the centromere on 21q have been implicated in maternally-derived trisomy 21 (Down syndrome) [32]. Our observation in spermatocytes may follow a similar pattern, where sperm from some infertile men may introduce an elevated risk of paternally-derived trisomy 21. Although altered crossover distribution as a risk factor for paternally derived trisomy 21 has not been extensively studied, Oliver *et al*. found weak evidence that an increase in crossovers near the centromere may play a role in paternal chromosome 21 nondisjunction [33].

Crossovers are also suppressed near the vicinity of the telomeres, possibly to prevent damage to the repetitive DNA [29]. Of the 16 infertile men with altered crossover distributions, five men displayed increased crossover formation near telomeres on one of the chromosomes studied. In *S*. *cerevisiae*, crossovers in this region may migrate to the ends of the chromosomes, disrupting the microtubule tension and lead to premature sister chromatid separation [34,35]. Potentially, an increase in crossovers near the centromeric or telomeric regions may disrupt the segregation of chromosomes and play a role in the production of aneuploid sperm in infertile men. Yet, this mechanism may be chromosome-specific as we only observed one infertile man with an altered crossover distribution on chromosome 13. In fact, the control group displayed a different pattern of crossover distribution on chromosome 13 compared to 18 and 21, where crossovers were most frequent at the centromere and telomere. It remains unclear whether chromosomes with a strong inhibition of crossovers at the centromere and telomeres are more prone to disruptions in crossover distribution in infertile men.

There has been evidence to suggest that discontinuities in the synapsis of homologous chromosomes may affect the crossover landscape in human males [36, 37]. In our study, four out of the seven NOA men, and five out of the nine OA men who showed altered crossover distributions also displayed increased rates of synaptic defects compared to controls. Although Sun *et al*. (2005) demonstrated that synaptic errors may have a *cis* effect on crossover distribution [36], this is unlikely to have influenced the altered crossover distributions seen in this study as we observed extremely low levels of unsynapsis on chromosomes 13, 18 and 21. However, it is possible that the synaptic errors in the infertile men had a *trans* effect on crossover patterns, similar to findings of a previous study [37].

### Increased crossover distance to telomeres in infertile men

In recent years, studies have shed light on the importance of telomeres in meiotic recombination. Telomere-guided mechanisms ensure sufficient DSB formation near the subtelomeres of the chromosomes in *S*. *cerevisiae* [29,38]. In normal males, increases in DSB activity and crossover formation have been observed near the subtelomeres compared to the rest of the chromosomes [2]. Our study demonstrated a decrease in crossover formation near subtelomeres in six of the 16 infertile men with altered crossover distributions. Moreover, the OA men as a group showed an altered crossover distribution with decreased crossover formation near subtelomeres on chromosome 18. Taking into account the average crossover distance to telomeres, the NOA and OA groups displayed increased distances on 21q and 18q respectively. These results may suggest a link between altered crossover distribution and compromised telomeres in infertile men, where this population shows reduced telomere length and impaired telomere integrity [14,39]. The copy number of telomeric or subtelomeric repeats could lead to changes in crossover positions [39]. Moreover, an absence of telomere-associated proteins that function in synapsis and DSB repair could reduce recombination rates [1,40–42]. Curiously, the NOA and OA groups did not show reduced crossover frequencies on the chromosomes with altered crossover distributions. This trend has been shown in *S*. *cerevisiae*, where the deletion of a telomere-associated protein, Tam1/Ndj1, altered the distribution, but not frequency of crossovers [43]. Tam1/Ndj1 mutants also displayed an increase in MI and MII nondisjunction, demonstrating the possible effect of impaired telomeres on aneuploidy [43]. Although there are limited studies on telomeric proteins in infertile men, a recent study reported an association between compromised telomere-associated proteins with reduced recombination [39].

### Altered crossover frequency and distribution in NOA and OA men

Our results provide further evidence that both NOA and OA men may have meiotic defects that affect the crossover positions but not frequencies [5,11]. Nonetheless, the degree of meiotic defects in NOA and OA men may be different, as the two types of severe infertility have distinct origins. NOA cases display impaired spermatogenesis in the testes, where a high degree of maturation arrest leads to the absence of sperm in the ejaculate. Although OA cases also lack sperm in the ejaculate, the phenotype is due to obstructions in the reproductive tract. From our data, two of the nine OA men, and five of the seven NOA men with altered crossover distributions showed altered crossover distribution and frequencies on the same chromosome. It seems that despite normal spermatogenesis, OA men may nevertheless possess meiotic defects in terms of crossover number and position. However, NOA men may be at a higher risk of having both alterations in the frequency and position of crossovers. Our findings align with previous data that suggested higher incidences of aneuploid sperm in NOA men compared to OA men [6,8]. Altered crossover frequency and distribution may have negative synergistic effects on chromosome segregation, leading to sperm with abnormal chromosome counts. Aside from the differing severity of meiotic defects seen in OA and NOA men, different mechanisms may be at play. Extended obstruction of the reproductive tract in OA cases may cause perturbations in the epigenetic regulation of DSB formation. Indeed, abnormal DNA methylation has been reported in the testicular tissues of OA men [44,45]. Future research on the epigenetic events associated with recombination, such as the trimethylation of histone H3 on lysine 4 (H3K4me3), and their correlation with crossover positions in OA men may elucidate the mechanisms behind the phenomenon [2,46]. Alternatively, meiotic defects in NOA cases may be due to genetic factors associated with impaired spermatogenesis. Mouse studies have shown that mutations in meiosis-specific genes such as *SCP3* and *MRE11*, which functions in DNA repair, may alter crossover distributions [47]. Variations in centromeric heterochromatin can also inhibit or promote crossovers, possibly resulting in the shift in crossovers toward the centromere [48]. Studies on azoospermic men have also identified mutations in *TEX11* and *TEX15*, which are genes shown to regulate recombination in mice [49–52]. Future work on the array of genes involved in synapsis and recombination may be useful for identifying the causes of the meiotic defects seen in the NOA population.

Overall, our results are in agreement with our previous findings, indicating that infertile men may possess meiotic defects that affect the frequency and distribution of crossovers. We confirmed several aberrant trends of crossover distribution in infertile men, such as the increased occurrence of crossovers near the centromere and telomeres, which may facilitate errors in chromosome segregation. On the other hand, small levels of variation in crossover distribution, as well as increases in crossovers near the middle of chromosome arms may not have repercussions on chromosome separation. Moving forward, we plan to investigate the relationship between crossover distribution and sperm aneuploidy in hopes of elucidating whether spermatocytes with abnormal crossover localization are arrested at meiotic checkpoints, or progress through meiosis, potentially giving rise to aneuploid sperm. Perhaps, substantial disturbances in the crossover landscape may be needed for detection by meiotic checkpoints, while smaller disparities in the crossover distribution may go unnoticed. In this case, it will also be interesting to correlate the severity of alteration in crossover distribution to the level of spermatogenic arrest, in addition to sperm aneuploidy in infertile men. Our study also provides valuable insight for future directions on the role of telomeres in crossover distribution, particularly the establishment of crossovers in subtelomeres. Finally, it is wise to consider the large variation in male infertility cases, where our sample size, although large, does not represent a homogenous group. For example, polymorphisms in *PRDM9*, which is an important determinant of recombination hotspots, have been shown to alter the crossover landscape [53–55]. Future work will employ genotyping techniques in order to identify which infertile sub-population may be most at risk of meiotic defects that contribute to adverse sperm outcome, and thus would benefit from genetic counselling prior to ICSI.

## References

[1] Joseph I, Lustig A. Telomeres in meiotic recombination: The Yeast Side Story. *Cellular and Molecular Life Sciences*. 2007;64:125–130. 17219024 10.1007/s00018-006-6464-1 PMC11136328

[2] Mézard C, Jahns MT, Grelon M. Where to cross? New insights into the location of meiotic crossovers. *Trends in genetics*: TIG. 2015;31:393–401. 10.1016/j.tig.2015.03.008 25907025

[3] Gonsalves J, Sun F, Schlegel PN, Turek PJ, Hopps CV, Greene C, et al Defective recombination in infertile men. *Hum Mol Genet*. 2004;13:2875–2883. 15385442 10.1093/hmg/ddh302

[4] Ma S, Ferguson K, Arsovska S, Moens P, Chow V. Reduced recombination associated with the production of aneuploid sperm in an infertile man: a case report. *HUMAN REPRODUCTION*. 2006; 2005;21:980–985. 16373411 10.1093/humrep/dei428

[5] Ferguson KA, Leung S, Jiang D, Ma S. Distribution of MLH1 foci and inter-focal distances in spermatocytes of infertile men. *Human Reproduction*. 2009;24:1313–1321. 10.1093/humrep/dep021 19246465

[6] Ferguson KA, Wong EC, Chow V, Nigro M, Ma S. Abnormal meiotic recombination in infertile men and its association with sperm aneuploidy. *Hum Mol Genet*. 2007;16:2870–2879. 17728321 10.1093/hmg/ddm246

[7] Sun F, Turek P, Greene C, Ko E, Rademaker A, Martin RH. Abnormal progression through meiosis in men with nonobstructive azoospermia. *Fertil Steril*. 2007;87:565–571. 17140569 10.1016/j.fertnstert.2006.07.1531

[8] Kirkpatrick G, Ferguson KA, Gao HJ, Tang S, Chow V, Ho Yuen B, et al A comparison of sperm aneuploidy rates between infertile men with normal and abnormal karyotypes. *Human Reproduction*. 2008;23:1679–1683. 10.1093/humrep/den126 18436578

[9] Hultén M, Eliasson R, Tillinger KG. Low chiasma count and other meiotic irregularities in two infertile 46, XY men with spermatogenic arrest. *Hereditas*. 1970;65:285 5525822 10.1111/j.1601-5223.1970.tb02327.x

[10] Mićić M, Mićić S, Diklić V. Low chiasma frequency as an aetiological factor in male infertility. *Clin Genet*. 1982;22:266–269. 7151310 10.1111/j.1399-0004.1982.tb01443.x

[11] Laurie DA, Hulten MA. Further studies on chiasma distribution and interference in the human male. Ann Hum Genet 1985;49:203–214. 4073834 10.1111/j.1469-1809.1985.tb01694.x

[12] Codina-Pascual M, Campillo M, Kraus J, Speicher MR, Egozcue J, Navarro J, et al Crossover frequency and synaptonemal complex length: their variability and effects on human male meiosis. *Mol Hum Reprod*. 2006;12:123–133. 16449239 10.1093/molehr/gal007

[13] Yang F, Silber S, Leu NA, Oates RD, Marszalek JD, Skaletsky H, et al TEX11 is mutated in infertile men with azoospermia and regulates genome‐wide recombination rates in mouse. *EMBO Molecular Medicine*. 2015;7:1198–1210. 10.15252/emmm.201404967 26136358 PMC4568952

[14] Thilagavathi J, Venkatesh S, Dada R. Telomere length in reproduction. *Andrologia*. 2013;45:289–304. 10.1111/and.12008 22928904

[15] Zickler D, Kleckner N. THE LEPTOTENE-ZYGOTENE TRANSITION OF MEIOSIS. *Annu Rev Genet*. 1998;32:619–697. 9928494 10.1146/annurev.genet.32.1.619

[16] Scherthan H. Telomeres and meiosis in health and disease: Telomere attachment and clustering during meiosis. *Cellular and Molecular Life Sciences*. 2007;64:117–124. 17219025 10.1007/s00018-006-6463-2 PMC11136177

[17] Scherthan H. A bouquet makes ends meet. *Nature Reviews Molecular Cell Biology*. 2001;2:621–627. 11483995 10.1038/35085086

[18] Ding X, Xu T, Xu R, Yu J, Zhuang Y, Han M. SUN1 Is Required for Telomere Attachment to Nuclear Envelope and Gametogenesis in Mice. *Developmental Cell*. 2007;12:863–872. 17543860 10.1016/j.devcel.2007.03.018

[19] Shibuya H, Ishiguro K, Watanabe Y. The TRF1-binding protein TERB1 promotes chromosome movement and telomere rigidity in meiosis. *Nat Cell Biol*. 2014;16:145–156. 10.1038/ncb2896 24413433

[20] DePinho RA, Greider CW, Horner JW, Lee H, Gottlieb GJ, Blasco MA. Essential role of mouse telomerase in highly proliferative organs. *Nature*. 1998;392:569–574. 9560153 10.1038/33345

[21] Zalenskaya I, Zalensky A. Telomeres in mammalian male germline cells In: Vol 218 SAN DIEGO: ELSEVIER ACADEMIC PRESS INC; 2002:37–67. 10.1016/s0074-7696(02)18011-9 12199519

[22] Keefe DL, Blasco MA, Liu L. Requirement of functional telomeres for metaphase chromosome alignments and integrity of meiotic spindles. *EMBO Rep*. 2002;3:230–234. 11882542 10.1093/embo-reports/kvf055 PMC1084019

[23] Liu L, Blasco MA, Trimarchi JR, Keefe DL. An Essential Role for Functional Telomeres in Mouse Germ Cells during Fertilization and Early Development. *Dev Biol*. 2002;249:74–84. 12217319 10.1006/dbio.2002.0735

[24] Liu L, Franco S, Spyropoulos B, Moens PB, Blasco MA, Keefe DL. Irregular Telomeres Impair Meiotic Synapsis and Recombination in Mice. *Proc Natl Acad Sci U S A*. 2004;101:6496–6501. 15084742 10.1073/pnas.0400755101 PMC404073

[25] Treff N, Su J, Taylor D, Scott R. Telomere DNA Deficiency Is Associated with Development of Human Embryonic Aneuploidy. *PLOS GENETICS*. 2011;7:e1002161 10.1371/journal.pgen.1002161 21738493 PMC3128107

[26] Lynn A, Koehler KE, Judis L, Chan ER, Cherry JP, Schwartz S, Seftel A, et al Covariation of Synaptonemal Complex Length and Mammalian Meiotic Exchange Rates. *Science*. 2002;296:2222–2225. 12052900 10.1126/science.1071220

[27] Tease C, Hultén MA. Inter-sex variation in synaptonemal complex lengths largely determine the different recombination rates in male and female germ cells. *Cytogenetic and Genome Research*. 2004;107:208–215. 15467366 10.1159/000080599

[28] Rockmill B, Voelkel-Meiman K, Roeder GS. Centromere-Proximal Crossovers Are Associated With Precocious Separation of Sister Chromatids During Meiosis in Saccharomyces cerevisiae. *Genetics*. 2006;174:1745–1754. 17028345 10.1534/genetics.106.058933 PMC1698618

[29] Blitzblau HG, Bell SP, Bell GW, Rodriguez J, Hochwagen A. Mapping of Meiotic Single-Stranded DNA Reveals Double-Strand-Break Hotspots near Centromeres and Telomeres. *Current Biology*. 2007;17:2003–2012. 18060788 10.1016/j.cub.2007.10.066

[30] Sun F, Oliver-Bonet M, Liehr T, Starke H, Turek P, Ko E, et al Variation in MLH1 distribution in recombination maps for individual chromosomes from human males. *Hum Mol Genet*. 2006;15:2376–2391. 16803849 10.1093/hmg/ddl162

[31] Reeves A. MicroMeasure: a new computer program for the collection and analysis of cytogenetic data. Genome 2001;44:439–443. 11444703

[32] Oliver T, Middlebrooks C, Tinker S, Allen EG, Bean LJ, Begum F, et al An Examination of the Relationship between Hotspots and Recombination Associated with Chromosome 21 Nondisjunction. *PLOS ONE*. 2014;9:e99560 10.1371/journal.pone.0099560 24926858 PMC4057233

[33] Oliver T, Bhise A, Feingold E, Tinker S, Masse N, Sherman S. Investigation of Factors Associated With Paternal Nondisjunction of Chromosome 21. *AMERICAN JOURNAL OF MEDICAL GENETICS PART A*. 2009;149A:1685–1690. 10.1002/ajmg.a.32942 19606484 PMC4111419

[34] Ross L.O., Rankin S., Flatters M., Dawson D. 1996 Effects of homology, size and exchange on the meiotic segregation of model chromosomes in *Saccharomyces cerevisiae*. Genetics 142: 79–89. 8770586 10.1093/genetics/142.1.79 PMC1206966

[35] Su Y, Barton AB, Kaback DB. Decreased meiotic reciprocal recombination in subtelomeric regions in Saccharomyces cerevisiae. *Chromosoma*. 2000;109:467–475. 11151676 10.1007/s004120000098

[36] Sun F, Oliver-Bonet M, Liehr T, Starke H, Trpkov K, Ko E, et al Discontinuities and unsynapsed regions in meiotic chromosomes have a cis effect on meiotic recombination patterns in normal human males. Hum Mol Genet 2005;14:3013–3018. 16155114 10.1093/hmg/ddi332

[37] Sun F, Oliver-Bonet M, Liehr T, Starke H, Ko E, Rademaker A, et al Discontinuities and unsynapsed regions in meiotic chromosomes have a trans effect on meiotic recombination of some chromosomes in human males. Cytogenet Genome Res 2007;119(1–2):27–32. 18160778 10.1159/000109615

[38] Peoples-Holst T, Burgess S. Multiple branches of the meiotic recombination pathway contribute independently to homolog pairing and stable juxtaposition during meiosis in budding yeast. *Genes Dev*. 2005;19:863–874. 15805472 10.1101/gad.1293605 PMC1074323

[39] Reig-Viader R, Capilla L, Vila-Cejudo M, Garcia F, Anguita B, Garcia-Caldes M et al Telomere homeostasis is compromised in spermatocytes from patients with idiopathic infertility. *Fertil Steril*. 2014;102:728–U434. 10.1016/j.fertnstert.2014.06.005 24996497

[40] Barton AB, Su Y, Lamb J, Barber D, Kaback DB. A function of subtelomeric DNA in Saccharomyces cerevisiae. Genetics 2003; 165:929–934. 14573499 10.1093/genetics/165.2.929 PMC1462788

[41] Watanabe Y, Cooper JP, Nurse P. Fission yeast Taz1 protein is required for meiotic telomere clustering and recombination. *Nature*. 1998;392:828–831. 9572143 10.1038/33947

[42] Wu H, Burgess SM. Ndj1, a Telomere-Associated Protein, Promotes Meiotic Recombination in Budding Yeast. *Mol Cell Biol*. 2006;26:3683–3694. 16648465 10.1128/MCB.26.10.3683-3694.2006 PMC1488995

[43] Chua PR, Roeder GS. Tam1, a telomere-associated meiotic protein, functions in chromosome synapsis and crossover interference. Genes & Dev. 1997;11:1786–1800. 9242487 10.1101/gad.11.14.1786

[44] Minor A, Chow V, Ma S. Aberrant DNA methylation at imprinted genes in testicular sperm retrieved from men with obstructive azoospermia and undergoing vasectomy reversal. *Reproduction (Cambridge, England)*. 2011;141:749–757. 10.1530/REP-11-0008 21389080

[45] Ferfouri F, Boitrelle F, Ghout I, Albert M, Molina Gomes D, Wainer R, et al A genome‐wide DNA methylation study in azoospermia. *Andrology*. 2013;1:815–821. 10.1111/j.2047-2927.2013.00117.x 23996935

[46] Kniewel R, Keeney S. Histone methylation sets the stage for meiotic DNA breaks. *EMBO J*. 2009;28:81–83. 10.1038/emboj.2008.277 19158660 PMC2634739

[47] Cherry SM, Adelman CA, Theunissen JW, Hassold TJ, Hunt PA, Petrini JHJ. The Mre11 Complex Influences DNA Repair, Synapsis, and Crossing Over in Murine Meiosis. *Current Biology*. 2007;17:373–378. 17291760 10.1016/j.cub.2006.12.048 PMC1839861

[48] Yamamoto M. INTERCHROMOSOMAL EFFECTS OF HETEROCHROMATIC DELETIONS ON RECOMBINATION IN DROSOPHILA MELANOGASTER. *Genetics*. 1979;93:437–448. 119672 10.1093/genetics/93.2.437 PMC1214091

[49] Ruan J, He X, Du W, Chen G, Zhou Y, Xu S, et al Genetic Variants in TEX15 Gene Conferred Susceptibility to Spermatogenic Failure in the Chinese Han Population. *Reproductive Sciences*. 2012;19:1190–1196. 10.1177/1933719112446076 22581801

[50] Yang F, Eckardt S, Leu NA, McLaughlin KJ, Wang PJ. Mouse TEX15 Is Essential for DNA Double-Strand Break Repair and Chromosomal Synapsis during Male Meiosis. *J Cell Biol*. 2008;180:673–679. 10.1083/jcb.200709057 18283110 PMC2265566

[51] Yang F, Silber S, Leu NA, Oates RD, Marszalek JD, Skaletsky H, et al TEX11 is mutated in infertile men with azoospermia and regulates genome‐wide recombination rates in mouse. *EMBO Molecular Medicine*. 2015;7:1198–1210. 10.15252/emmm.201404967 26136358 PMC4568952

[52] Zhang X, Ding M, Ding X, Li T, Chen H. Six polymorphisms in genes involved in DNA double-strand break repair and chromosome synapsis: association with male infertility. *Systems Biology in Reproductive Medicine*. 2015;61:187–193. 10.3109/19396368.2015.1027014 26086992

[53] Baudat F, Buard J, Grey C, Fledel-Alon A, Ober C, Przeworski M, et al PRDM9 Is a Major Determinant of Meiotic Recombination Hotspots in Humans and Mice. *Science*. 2010;327:836–840. 10.1126/science.1183439 20044539 PMC4295902

[54] Berg IL, Neumann R, Sarbajna S, Odenthal-Hesse L, Butler NJ, Jeffreys AJ. Variants of the protein PRDM9 differentially regulate a set of human meiotic recombination hotspots highly active in African populations. *Proc Natl Acad Sci U S A*. 2011;108:12378–12383. 10.1073/pnas.1109531108 21750151 PMC3145720

[55] Myers S, Bowden R, Tumian A, Bontrop RE, Freeman C, Macfie TS, et al Drive against Hotspot Motifs in Primates Implicates the PRDM9 Gene in Meiotic Recombination. *Science*. 2010;327:876–879. 10.1126/science.1182363 20044541 PMC3828505

